# The effect of oral capsules containing *Ocimum basilicum* leaf extract on menopausal symptoms in women: a triple-blind randomized clinical trial

**DOI:** 10.1186/s40001-024-01965-7

**Published:** 2024-07-16

**Authors:** Fatemeh Zahra karimi, Hamideh Hosseini, Seyed reza Mazlom, Hassan Rakhshandeh

**Affiliations:** 1 Nursing and Midwifery Care Research Center, Mashhad University of Medical Sciences, Mashhad, Iran; 2 Department of Midwifery, School of Nursing and Midwifery, Mashhad University of Medical Sciences, Mashhad, Iran; 3 Department of Midwifery, School of Nursing and Midwifery, Birjand University of Medical Sciences, Birjand, Iran; 4 Department of Pharmacology, Medical Plants Pharmacological Research Center, Faculty of Medicine, Mashhad University of Medical Sciences, Mashhad, Iran

**Keywords:** Menopausal symptoms, *Ocimum basilicum*, Women, Menopause

## Abstract

**Background:**

Menopause, characterized by various physical and mental changes, is primarily caused by hormonal fluctuations, resulting in numerous complications. Recently, herbal treatments have gained significant attention for their minimal side effects compared to chemical drugs. This study aimed to investigate the effects of oral capsules containing *Ocimum basilicum* leaf extract (OBLE) on menopausal symptoms.

**Methods:**

This placebo-controlled clinical trial study was conducted in 2020. The research focused on 60 menopausal women referred to Mashhad health centers. Eligible participants were administered either an OBLE 500 mg capsule or a placebo daily for 1 month. Menopause symptoms were evaluated using the Menopause Rating Scale (MRS) before, two weeks, and one month after the intervention. Data were analyzed using SPSS21, independent t, Mann–Whitney, and Friedman tests. A significance level of *p* < 0.05 was considered significant.

**Results:**

The independent t-test indicated that the mean (SD) scores of menopausal symptoms in both the OBLE and placebo groups were initially similar before the intervention (*P* = 0.141). Two weeks after the intervention, the menopausal symptom scores were 9.5 ± 3.5 and 11.2 ± 5.6 in the OBLE and placebo groups, respectively (*P* = 0.163, *df* = 58, *t* = 1.4). After one month, the menopausal symptom scores were 6.9 ± 0.3 in the OBLE group and 11.26 ± 0.6 in the placebo group (*P* = 0.001, *df* = 58, *t* = 3.4). This indicates a significant difference between the two groups one month after the intervention, compared to before and two weeks after the intervention. Additionally, there was a significant difference in the scores of the physical and somato-vegetative dimension between the intervention and placebo groups two weeks and one month after the intervention (*P* < 0.05).

**Conclusion:**

The study results suggested that taking OBLE capsules led to a decrease in the scores of menopausal symptoms. This indicates that OBLE can be considered as a safe and cost-effective medicinal plant for alleviating menopausal symptoms among women.

## Introduction

Menopause, a significant phase in women’s lives (1), affects approximately 95% of women worldwide as life expectancy continues to rise (2). In Iran, the 2015 census estimated that nearly 7 million women fell within the 45–65 age group (3). Hormonal changes during menopause can lead to various complications, including hot flashes, sleep disturbances, vaginal dryness, and mood changes (4–6). These symptoms are experienced by approximately 65–85% of women during menopause (7).

Common problems during menopause are hot flashes, vasomotor disorders, changes in the reproductive system, skin changes, cardiovascular diseases, and osteoporosis, respectively (8).

Vasomotor symptoms occur in 80% of menopausal women, with 25% experiencing severe intensity. Vaginal symptoms, such as vaginal dryness and dyspareunia, affect about one-third of postmenopausal women, and can last for years or start several years after menopause (7). Sleep disturbances and depressive symptoms are experienced by about one-third and 10% of postmenopausal women, respectively (9).

Menopause symptoms are highly prevalent and cause significant concern, with approximately 90% of women seeking ways to cope with them (10).

While hormone therapy is commonly used to alleviate these symptoms (11), concerns regarding its potential risks such as endometrial and breast cancer, heart attacks, and vaginal bleeding, have led to restrictions on its use for certain women (12). Additionally, some women prefer to avoid hormone therapy and instead seek complementary and alternative medicines to manage their menopausal symptoms (13).

Complementary and alternative medicines, such as behavioral therapy, yoga, stress management, nutritional supplements, and herbal medicine, are commonly used to alleviate menopausal symptoms (13). Among these approaches, herbal medicine is particularly popular due to its lower costs and fewer side effects compared to hormone therapy (14). The World Health Organization (WHO) recognizes the use of complementary and herbal medicines as a means to prevent and relieve menopausal symptoms (15).

Herbal medicines containing phytoestrogens, which are compounds with estrogenic properties, are commonly used to alleviate menopausal symptoms (16). Phytoestrogens encompass various compounds such as flavonoids, isoflavonoids, stilbenes, and lignans (17). Additionally, phytosterols, a group of steroids, act as selective estrogen receptor modulators (SERMs) (18). In a study by Motaghi Dastanai et al. (2017), the effect of evening primrose plant on the physical symptoms of menopause was examined, showing improvement in physical symptoms due to its phytoestrogenic properties (19). Similarly, in the study conducted by Aftan et al. (2023), soybean oil and evening primrose oil were effective in improving menopause symptoms due to phytoestrogens (20).

Ocimum basilicum (OB), a herb belonging to the Lamiaceae family, is known for its phytoestrogenic properties (21). It is native to Iran and India (22). In traditional Iranian medicine, OB is used to address symptoms such as anxiety, depression, fatigue, and insomnia (21).

Basil essential oil is composed of various compounds, including estragole, linalool, eugenol, methyl chavicol, cineole, as well as monoterpenes, sesquiterpenoids, flavonoid ethyl acetate, and rosmarinic acid (23). A study by Nagib (2019) investigated the effects of basil powder and extract oil on menopausal symptoms in female mice, showing positive effects due to its phenolic compounds (24). The safety of basil has also been confirmed in a study conducted by Askari et al. in 2016 (25). Basil’s composition includes flavonoids such as quercetin and luteolin, as well as phytosterols like beta-sitosterol, suggesting its potential as a selective estrogen receptor modulator (estrogen modulator) in menopausal women, providing relief from menopausal symptoms (25, 26). A review study by Miraj and Kiani (2016) investigated the pharmacological effects of OB, including anti-osteoporotic, anxiolytic and sedative, antihypertensive, antithrombotic, antioxidant, antihypertensive, vasorelaxant, and anti-platelet effects (27).

Menopause symptoms can have negative effects on women’s well-being. Previous research on the compounds found in *Ocimum basilicum* (OB) and an animal study suggest its potential in alleviating menopausal symptoms. Due to the lack of clinical studies examining the effects of OB on menopausal symptoms, and the preference of many women for herbal medicine as an alternative to hormone therapy, this study aimed to assess the impact of oral capsules containing *Ocimum basilicum* leaf extract (OBLE) on menopausal symptoms.

## Methods

This triple-blind, randomized clinical trial was conducted on 60 menopausal women who were attending health centers in Mashhad from February 9th to September 4th, 2020. The study was approved by the Ethics Committee of Mashhad University of Medical Sciences (IR.MUMS.NURSE.REC.1398.070) and registered in the Iranian Registry of Clinical Trials (IRCT20200104046001N1). The inclusion criteria were as follows: menopausal women aged between 40 and 65, who had ability to read and write, were living in Mashhad, had no history of mental illness, no hormone therapy within the last 6 months, no accidents within the last 6 months, and no smoking or alcohol consumption.

The exclusion criteria included failure to take medication correctly^1^ occurrence of a stressful incident during the study, drug sensitivity^2^ hormone therapy during the study, and use of herbal medicines containing phytoestrogens during the study^3^

The sample size was based on a pilot study conducted on 10 individuals in each group within the same community. The mean (SD) scores for the OBLE capsule group and the placebo group were 7.3 ± 3.6 and 9.7 ± 2.8, respectively. With a confidence interval of 95% and a test power of 80%, the initial sample size was 29 women in each group. However, to account for a 30% dropout rate, the sample size was increased to 38 individuals in each group, resulting in a total of 76 women. After excluding 16 individuals, the final sample consisted of 60 women.

The study used several tools, including a case selection checklist, a personal characteristics questionnaire, and the Menopause Rating Scale (MRS). The personal characteristics questionnaire encompassed various aspects such as demographics, gynecological history, and information pertaining to menopause. These tools were used to gather relevant data and assess the participants’ personal characteristics and experiences related to menopause.

The MRS comprises 11 symptoms categorized into three dimensions: physical (hot flashes and night sweats, heart problems, sleep disturbance, physical fatigue, and muscle and joint pain), psychological (depressed mood, irritability, anxiety, and mental fatigue), and urinary-genital (sexual problems, urinary problems, and vaginal dryness). Each question is rated on a 5-point Likert scale ranging from 0 (no symptoms) to 4 (very severe).

The ranges of scores for the psychological, physical, and urinary-genital dimensions are 0–16, 0–16, 0–12, respectively. According to this tool, lower scores indicate fewer menopause symptoms. Heinemann et al. (2002) confirmed the validity, reliability, and applicability of this tool for the first time, with a correlation coefficient of 0.82 (28). Additionally, the validity and reliability of this tool in Iran were confirmed by Darsareh et al. in 2012 (29). In the present study, the reliability was assessed using the internal-consistency method, resulting in a Cronbach’s alpha coefficient of 0.72.

Data were collected from five primary healthcare centers in Mashhad, with centers No. 1 and 3 randomly selected. From each of these two centers, two comprehensive health sub-centers were chosen based on the higher number of menopausal women referrals. Convenience sampling was employed for selecting the cases.

After explaining the study objectives and obtaining informed consent from the participants, the case selection process was completed. Those who met the study criteria proceeded to complete the MRS questionnaire. Menopausal women were then randomly assigned to two groups: one receiving OBLE capsules and the other receiving a placebo. The random allocation was determined using a sequence generated by the website (www.randomization.com). Cards were written and placed in sealed envelopes according to the generated sequence. Participants would open the envelope to determine their assigned group. To maintain triple-blind conditions, neither the researchers, participants, nor the statistical analyst were aware of the group assignments or the type of capsules (OBLE or placebo). The capsule packages were coded with A and B by the pharmacist. Participants in group A received capsules with code A, while participants in group B received capsules with code B. Thus, after opening the envelope and determining their groups, participants were provided with the corresponding capsules. In this study, neither the participants, researchers, nor statistical analysts were aware of the capsule contents, and only the pharmacist who produced and packaged the capsules knew the contents, which were determined after the study and statistical analysis.

To prepare the medication, we obtained washed and dried OB leaves from an organic farm affiliated with Ferdowsi University of Mashhad. These leaves were verified by a botanist at the herbarium department in the Faculty of Pharmacy in Mashhad (herbarium code: 12,937 obl). The leaves were then ground into a fine powder and soaked in a 70% hydroalcoholic solution for 3–5 days. After removing the solvent and filtering, the resulting hydroalcoholic extract was combined with Avicel to form a powder. This powder was then encapsulated in 500 mg capsules. Starch capsules of the same weight were used for the placebo, which were indistinguishable from the OB capsules.

During the study, participants were instructed to take one capsule every night for one month. To ensure compliance and monitor any potential complications, the researcher maintained regular communication with the participants. Weekly phone calls or text messages were used to confirm correct medication intake, inquire about complications, and assess overall satisfaction. Additionally, a meeting was scheduled a few days before the end of the study, during which the participants completed the Menopause Rating Scale (MRS) twice: two weeks and one month after the intervention.

Data gathered from the study were analyzed using SPSS21. Descriptive statistics, such as frequency tables, mean, and standard deviation, were employed to describe the characteristics of the cases. The normality of the data was assessed with the Kolmogorov–Smirnov test. For normally distributed data, an independent t-test was utilized, while non-normal data were analyzed using the Mann–Whitney and Friedman tests. Nominal variables were examined using the Chi-square and Fisher’s exact tests. A significance level of *p* < 0.05 was considered significant.

## Results

**Figure1:**
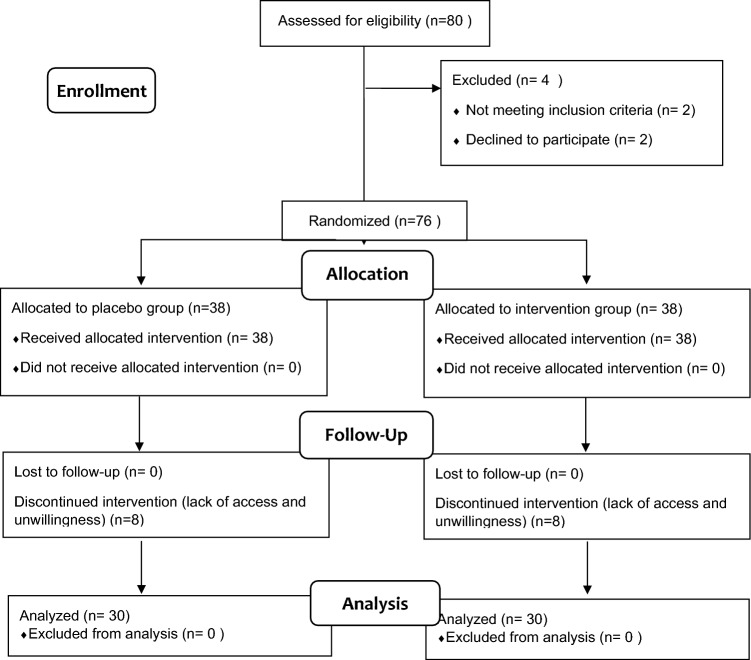
The process of study design

A total of 16 participants, 8 from the intervention group and 8 from the placebo group, were excluded from the study (Fig. 1). As a result, the study was conducted with 30 patients in each group. Both the OBLE supplement and placebo were well tolerated, and no serious side effects were observed during the study period.

In this study, the mean age of women in the OBLE group was 55.7 ± 5.7, while in the placebo group it was 56.6 ± 6.1. Other demographic information did not show significant differences between the OBLE and placebo groups, indicating that the two groups were homogeneous (*p* > 0.05) (Table 1).

**Table 1:** Qualitative analysis of demographic variables in the OBEL and placebo groups

Test result	Group			Variables
	OBLE(*n* = 30)	Placebo(*n* = 30)		
	SD ± mean	SD ± mean		
0.591 = P^a^58, = df 0.5, = t	5.7 ± 55.7	6.1 ± 56.6		Age (years)
0.314 = P^a^58, = df 1.0, = t	5.4 ± 27.6	3.7 ± 26.3		Body mass index (kg/m^2^)
0.658 = P^a^58, = df 0.5, = t	5.7 ± 7.4	5.9 ± 8.1		Duration of menopause (years)
0.621 = P^a^58, = df 0.5, = t	2.0 ± 3.7	1.5 ± 3.4		Gravidity
0.809 = P^a^58, = df 0.2, = t	1.8 ± 3.2	1.3 ± 3.1		Para
0.933 = P^a^58, = df 0.1, = t	1.7 ± 3.1	1.4 ± 3.0		Child
	Frequency (percentage)	Frequency (percentage)		
1.4 = Z 0.158 = P^b^	(16.7)5	(13.3) 4	Elementary	Education
	(26.7) 8	(10.0) 3	Under diploma	
	(30.0) 9	(36.7) 11	Diploma	
	(26.7) 8	(40.0) 12	Academic	
3 = df ، 2.3 = Chi^2^ 0.527 = P^c^	(63.3) 19	(60.0) 18	House wife	Job
	(13.3) 4	(26.7) 8	Self-employed	
	(13.3) 4	(6.7) 2	Employee	
	(10.0) 3	(6.7) 2	Retired	
0.554 = P^c^	(3.3) 1	(6.7) 2	Yes	History of hormone therapy (to relieve menopausal symptoms)
	(96.7) 29	(93.3) 28	No	

^a^Independent T-test, ^b^aMann–Whitney, ^c^exact Chi-square test

Before the intervention, the total mean (SD) scores for menopausal symptoms in the OBLE and placebo groups were 12.7 ± 4.9 and 10.8 ± 4.9, respectively. An independent t-test did not reveal a significant difference between the two groups (*p* = 0.141).

The independent t-test was used to compare the total scores of menopausal symptoms in both groups at two weeks and one month after the intervention. The results indicated no significant difference in the total scores of menopausal symptoms between the two groups two weeks after the intervention (*p* = 0.163).

However, the results revealed that the total score of menopausal symptoms in the OBLE group was significantly lower than that of the placebo group after one month (*p* = 0.001).

Based on within-group comparison, only the mean score of menopausal symptoms in the OBLE group showed a significant decrease after one month compared to the pre-intervention phase (*p* < 0.001) (Table 2).

**Table 2:** Mean score of menopausal symptoms in OBEL and placebo groups before and 2 weeks and 1 month after the intervention

Inter-group test result	Group		Menopausal symptoms
	Placebo (*n* = 30)	OBLE (*n* = 30)	
	SD ± mean	SD ± mean	
*P* = 0.141^a^ ،58 = *df* ، 1.4 = *t*	10.4 ± 8.9	12.4 ± 7.9	Before intervention
*P* = 0.163^a^ ،58 = *df* ، 1.4 = *t*	11.5 ± 2.6	9.3 ± 5.5	2 weeks after intervention
*P* = 0.001^a^ ،58 = *df* ، 3.4 = *t*	11.0 ± 26.6	6.3 ± 9.0	1 month after intervention
	2 = *df* ، 3.6 = *Chi2* P = 0.159^b^	2 = *df* ، 38.9 = *Chi2* 0.001 > *P*^b^	Intra-group test result

^a^Independent T-test, ^b^Friedman

The post hoc test results indicated significant differences in various time periods related to the intervention. Specifically, there were significant differences before (*p* < 0.001), one month (*p* < 0.001), and two weeks after the intervention (*p* < 0.001).

In addition to the total score of menopausal symptoms, the study also examined the dimensions of menopausal symptoms, including psychological, somato-vegetative, and urogenital scores. Among these dimensions, there were significant differences in two dimensions, namely psychological and somato-vegetative scores, between the OBLE and placebo groups one month after the intervention (*p* < 0.05).

However, there were no significant differences in the urogenital dimension of menopausal symptoms between the OBLE group and the placebo group two weeks (*p* = 0.620) and one month after the intervention (*p* = 0.114) (Tables 3, 4, and 5).

**Table 3:** Psychological score of menopausal symptoms in OBEL and placebo groups before and 2 weeks and 1 month after the intervention

Inter-group test result	Group		Psychologic score
	Placebo (*n* = 30)	OBLE (*n* = 30)	
	SD ± mean	SD ± mean	
*P* = 0.141^a^ ،58 = *df* ، 1.4 = t	4.0 ± 2.3	5.3 ± 2.3	Before intervention
*P* = 0.163^a^ ،58 = *df* ، 1.4 = t	4.4 ± 2.4	3.9 ± 1.9	2 weeks after intervention
*P* = 0.001^a^ ،58 = *df* ، 3.4 = *t*	4.5 ± 2.5	2.8 ± 1.6	1 month after intervention
	2 = df ، 2.5 = *Chi2* P = 0.285^b^	2 = *df* ، 34.4 = Chi2 0.001 > *P* **	Intra-group test result

^a^Independent T-test,^b^Friedman

**Table 4:** Somato-vegetative score of menopausal symptoms in OBEL and placebo groups before and2 weeks and 1 month after the intervention

Inter-group test result	Group		Somato-vegetative
	Placebo (*n* = 30)	OBLE (*n* = 30)	
	SD ± mean	SD ± mean	
*P* = 0.693^a^ ،*z* = 0.3	3.3 ± 2.5	3.6 ± 2.4	Before intervention
*P* = 0.337^a^ ،*z* = 0.9	3.7 ± 2.7	2.9 ± 1.7	2 weeks after intervention
*P* = 0.009^a^ ،*z* = 2.6	3.9 ± 2.8	2.2 ± 1.5	1 month after intervention
	2 = df ، 8.0 = Chi2 P = 0.018^b^	2 = df ، 24.3 = Chi2 0.001 > P^b^	Intra-group test result

^a^Mann–Whitney, ^b^Friedman

**Table 5:** Urogenital score of menopausal symptoms in OBEL and placebo groups before and 2 weeks and 1 month after the intervention

Inter-group test result	Group		Urogenital
	Placebo (n = 30)	OBLE (n = 30)	
	SD ± mean	SD ± mean	
*P* = 0.59^a^ ،58 = *df* ، −0.541 = t	3.4 ± 2.1	3.7 ± 2.1	Before intervention
*P* = 0.620^b^ ،*z* = 0.4	3.3 ± 2.2	2.7 ± 1.7	2 weeks after intervention
*P* = 0.114^b^ ،*z* = 1.5	2.8 ± 2.2	1.8 ± 1.4	1 month after intervention
	2 = *df* ، 1.4 = *Chi2* *P* = 0.481^c^	2 = *df* ، 22.1 = *Chi2* 0.001 > *P*^*c*^	Intra-group test result

^a^Independent T-test, ^b^Mann–Whitney, ^c^Friedman

## Discussion

This study indicated a significant difference in the total scores of menopause symptoms between the OBLE capsule and placebo groups one month after the intervention. The basil capsule group experienced less severe menopause symptoms.

Complications arising from hormonal changes greatly affect the quality of life of menopausal women (4–6, 30). While hormone therapy is commonly used to alleviate these symptoms, it carries risks such as increased chances of heart attack, vaginal bleeding, endometrial and breast cancer (11, 12). As a result, herbal medicines have gained attention due to their affordability and lower side effects when compared to hormone therapy (17). Herbal remedies containing estrogenic compounds, known as phytoestrogens, are often used to alleviate menopause symptoms (16, 31).

OB essential oil is composed of various compounds, including estragole, linalool, eugenol, methyl chavicol, cineole, and basil. Basil itself contains a range of compounds such as monoterpenes, sesquiterpenoids, flavonoids, ethyl acetate, and rosmarinic acid (23). Nagib (2019) investigated the effects of basil powder and extract oil on menopausal symptoms in female rats. Their study revealed that the presence of phenolic compounds in basil contributed to the alleviation of menopause symptoms (24).

Basil’s composition also includes flavonoids like quercetin and luteolin, as well as phytosterols such as beta-sitosterol. These components enable basil to act as a selective estrogen receptor modulator (estrogen modulator) in menopausal women, thereby providing relief from menopause symptoms (25, 26).

In the study by Abdali et al. (2016), the effects of hypiran and fennel, two plants containing phytoestrogen, on menopause symptoms were examined. The results showed that fennel and hypiran were effective in improving menopausal signs and symptoms in women (32).

Plants containing phytoestrogens have frequently been used to improve menopausal symptoms. However, in the study by Sahraian et al. (2023), the consumption of foods containing phytoestrogens was not effective in alleviating the physical symptoms of menopause, which is not consistent with the results of the present study (8). This discrepancy in results can be due to nutritional differences in the research community and the use of phytoestrogens in medicinal forms, such as OB capsules, which provide a controlled and accurate dose to menopausal women.

Other herbs that contain similar compounds to OB have been studied and evaluated for their potential in alleviating menopause symptoms in women. One such herb is sage. Dadfar et al. (2019) examined the efficacy of *Salvia officinalis* extract oil. Prior to the intervention, there was no statistically significant difference in the mean scores of menopause symptoms between the lavender and placebo groups. However, after the intervention, a statistically significant difference in the mean scores of menopause symptoms was observed between the two groups, with the intervention group experiencing a lower mean score. The researchers concluded that sage extract oil, which contained phytoestrogens, was effective in relieving menopause symptoms (33).

Lemon balm, belonging to the Lamiaceae family, contains compounds like geraniol, linalool, rosmarinic acid, and flavonoids. Taavoni (2016) investigated the impact of lemon balm on menopause symptoms in women over an 8-week period. Initially, there was no statistically significant difference in the mean scores of menopause symptoms between the lemon balm and placebo groups. However, one and two months after the intervention, the lemon balm group experienced a significant reduction in the mean score of menopause symptoms. Lemon balm also had a positive effect on both physical and mental dimensions; although, no significant difference was observed in the urinary-genital dimension between the two groups (34).

Based on the results of these studies, phenolic compounds found in the Lamiaceae family, including lemon balm, may effectively alleviate menopause symptoms.

Menopause is often accompanied by symptoms such as irritability, anger and depression (35). OB also has sedative and anti-depressant properties (36). In a study by Mustafapour et al. (2018), the use of basil hydroalcoholic extract reduced depression in mice (37).

### Study limitation

Random allocation and triple-blindness are considered as strengths of the present study. However, it is important to acknowledge that the limitation of this research lies in the use of self-reported questionnaires.

## Conclusions

The consumption of OBLE oral capsules has been found to reduce the overall severity of menopause symptoms in women. This makes OBLE capsules a convenient and affordable option. Additionally, unlike chemical drugs, OBLE capsules do not have any adverse effects. Therefore, it is recommended as a means to alleviate menopause symptoms in women.

## Data Availability

The datasets used and analyzed during the current study are available from the corresponding author on reasonable request.

## Abbreviation

WHO: The World Health Organization
SERMs: Selective estrogen receptor modulators
OB: *Ocimum basilicum*
OBLE: *Ocimum basilicum* Leaf extract
MRS: Menopause rating scale

## Footnotes

1 Two consecutive days or four intermittent days.

2 Itching, inflammation, urticaria, skin redness, abdominal pain, diarrhea, nausea, and vomiting.

3 Lavender, Lemon Balm, Black Cohosh, Red Clover, Salvia Officinalis, *Glycyrrhiza glabra*, Basil Tea.
